# Supplementary material for: Bee-pollination promotes rapid divergent evolution in plants growing in different soils
Source: Nat Commun. 2024 Mar 27;15:2703. doi: 10.1038/s41467-024-46841-4 (PMC10973342; doi:10.1038/s41467-024-46841-4)
Supplement: Supplementary file 1 — Supplementary Information [file 41467_2024_46841_MOESM1_ESM.pdf]

Bee-pollination promotes rapid divergent evolution in plants growing in different soils

Thomas Dorey[1,2] and Florian P. Schiestl[1*]

[1]Department of Systematic and Evolutionary Botany, University of Zürich, Switzerland

[2] present address: Department of Environmental Sciences, University of Basel, Switzerland

*Corresponding author: florian.schiestl@systbot.uzh.ch

Supplementary Information

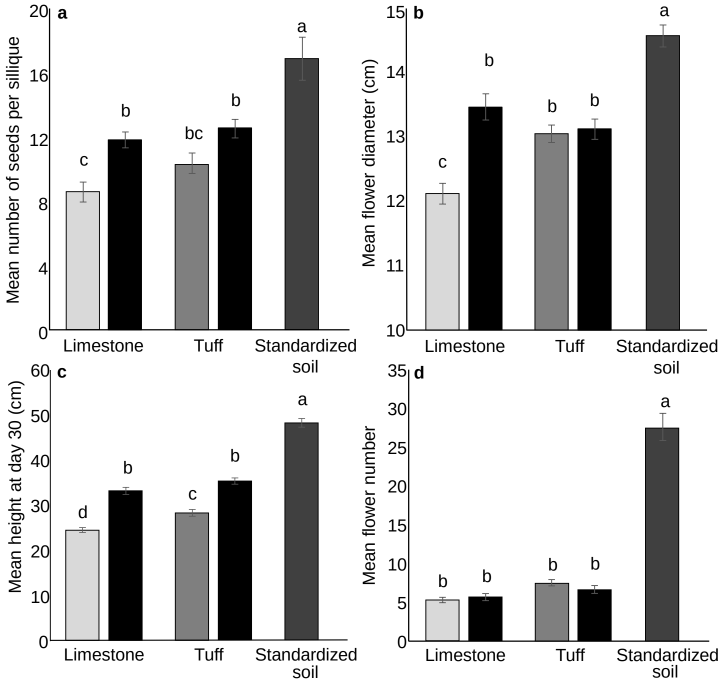

Supplementary Figure 1: Impact of soil type on plants' reproduction and phenotype (mean ± s.e.m. values). a: on number of seeds per silique (N=421), b: on flower diameter (N=399), c: on plant height at day 30 (N=412), d: on flower number at pollination date (N=412). For limestone and tuff soil, the left bar shows the soil without fertilizer, and the right bar with fertilizer (i.e. the soil type used in the experiment starting from generation 5). The standardized soil is the soil used for growing plants in the common garden experiment for phenotyping plants. Significance was determined using a two-sided generalized mixed model with trait as dependent variable, "soil treatment" and their interaction as fixed factors (5 levels: limestone with fertilizer, limestone without fertilizer, tuff with fertilizer, tuff without fertilizer, and standardized soil) and replicate as random factors. Different letters indicate significant differences between treatments ($P<0.05$). We detected resource limitation in seed production in

hand-pollinated plants growing in both natural soils with or without fertilizer, as the use of standardized soil always increased seed set in plants (effect of "soil treatment", χ^2: 57.13, P<0.001). The data also show that the plants' trait development were different after the use of fertilizer for both soil types, with significant increases in flower diameter and plant height, but overall still smaller/lower trait production that with standardized soil even with the use of fertilizer (effect of "soil treatment", χ^2: 608.79, P<0.001, χ^2: 524.68, P<0.001, χ^2: 118.34, P<0.001 for height day at 30, number of open flowers and flower diameter, respectively).

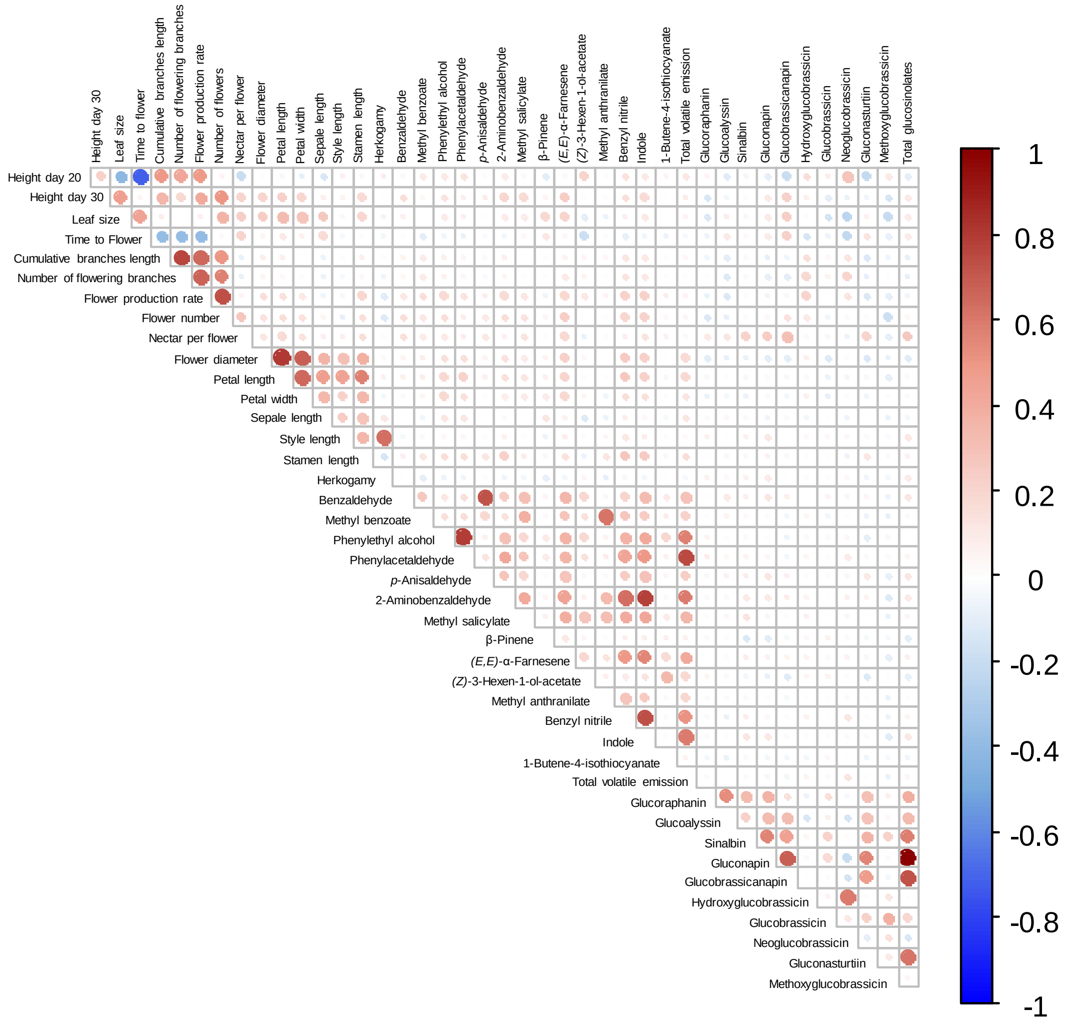

Supplementary Figure 2: Pearson product-moment correlation heatmap using plants of all generations and treatments combined (N=515). Nectar, scent and glucosinolates variables were ln (1+x) transformed before correlations were calculated. Positive correlations are shown in shades of red, and negative correlation in shades of blue. The size of the circle is proportional to the correlation coefficients. Only significant correlations (P<0.05) are shown.

Supplementary Table 1: Evolutionary rates assessed in haldanes (s.d. per generation) for plant traits among the different evolutionary lines. LHB: plants that evolved in limestone with aphid-herbivory and bee-pollination, LHH: plants that evolved in limestone with aphid-herbivory and hand-pollination, LNHB: plants that evolved in limestone with no-herbivory and bee-pollination, LNHH: plants that evolved in limestone with no-herbivory and hand-pollination, THB: plants that evolved in tuff with aphid-herbivory and bee-pollination, THH: plants that evolved in tuff with aphid-herbivory and hand-pollination, TNHB: plants that evolved in tuff with no-herbivory and bee-pollination, TNHH: plants that evolved in tuff with no-herbivory and hand-pollination. Bold indicates evolutionary changes in traits that had significantly different mean values between generation 10 and generation one.

	LHB	LHH	LNHB	LNHH	THB	THH	TNHB	TNHH
Morphology								
Height day 20 (cm)	0.00	-0.02	-0.02	-0.02	**-0.12**	0.00	**-0.07**	-0.02
Height day 30 (cm)	**-0.06**	0.00	**-0.05**	-0.02	-0.02	-0.02	**0.07**	0.02
Flowering time (day)	0.00	**0.05**	0.02	**0.05**	**0.11**	0.02	**0.11**	**0.03**
Leaf size (cm2)	-0.02	0.02	0.01	0.02	**0.08**	-0.01	**0.11**	0.04
Cumulative branches length (cm)	0.00	0.01	0.00	**-0.03**	**-0.02**	0.02	0.01	0.00
Number of flowering branches	0.02	0.02	0.02	-0.02	-0.01	0.00	-0.01	0.01
Flower production rate (per day)	0.00	**0.07**	0.02	0.04	**0.11**	0.01	**0.10**	**0.07**
Flower number	0.00	**0.05**	0.03	0.03	**0.06**	0.00	**0.07**	**0.06**
Nectar amount (nL/flower)	-0.01	0.03	-0.01	0.01	**0.04**	**0.04**	**0.07**	**0.06**
Flower diameter (mm)	0.02	**-0.03**	**0.03**	-0.01	**0.04**	-0.01	**0.05**	**-0.04**
Petal length (cm)	0.01	-0.03	0.01	-0.01	**0.03**	-0.01	0.03	**-0.04**
Petal width (cm)	0.02	**-0.05**	**0.05**	0.01	0.01	-0.03	0.02	0.00
Sepale length (cm)	**-0.04**	**-0.04**	0.01	0.00	-0.01	-0.01	0.01	-0.03
Style length (cm)	-0.03	-0.01	-0.03	-0.02	-0.02	0.00	-0.01	**-0.05**
Stamen length (cm)	-0.02	-0.02	-0.03	-0.02	0.03	-0.01	-0.02	0.01
Herkogamy	-0.01	0.01	-0.01	-0.01	0.00	0.02	0.00	-0.03
Floral Scent								
**Aromatic compounds**								
Benzaldehyde	0.00	-0.03	0.02	**0.04**	**0.04**	**0.07**	**0.05**	0.02
Methyl benzoate	**0.05**	-0.01	0.00	0.00	**-0.03**	0.00	0.01	**-0.05**
Phenylethyl alcohol	0.01	0.02	**0.04**	**0.07**	-0.03	0.00	-0.01	0.01
Phenylacetaldehyde	0.02	0.01	0.03	**0.09**	**-0.05**	0.00	-0.02	-0.02
p-Anisaldehyde	0.01	0.00	0.03	0.02	0.03	**0.03**	0.02	-0.02
2-Aminobenzaldehyde	0.01	**0.03**	-0.02	0.02	-0.02	0.00	0.01	-0.02
Methyl salicylate	0.03	0.00	-0.01	0.02	0.00	-0.02	0.02	-0.03
**Terpenoids**								
β-Pinene	-0.03	-0.02	-0.02	-0.03	-0.02	-0.03	-0.01	-0.02
(E.E)-α-Farnesene	-0.02	-0.01	0.02	0.03	0.03	0.03	**0.05**	-0.01
**Fatty acid derivatives**								
(Z)-3-Hexen-1-ol-acetate	0.01	-0.01	0.01	0.00	**-0.05**	0.02	0.02	-0.01
**Nitrogen containing compounds**								
Methyl anthranilate	**0.04**	0.02	**-0.06**	0.00	**-0.07**	**-0.04**	-0.03	**-0.08**
Benzyl nitrile	**0.06**	0.01	**0.03**	0.03	0.00	0.01	0.00	-0.01
Indole	**0.04**	**0.03**	0.00	0.03	0.02	0.01	0.03	0.00

**Sulphur containing compounds**								
1-Butene-4-isothiocyanate	-0.01	0.00	0.02	**0.04**	0.00	0.03	0.02	0.03
Leaf glucosinolates								
Glucoraphanin	-0.02	0.00	**-0.05**	0.01	**-0.04**	0.01	**-0.05**	-0.03
Glucoalyssin	**-0.04**	-0.01	0.03	0.03	0.02	0.01	0.00	0.01
Sinalbin	-0.02	-0.02	-0.03	-0.01	0.00	0.00	-0.01	-0.01
Gluconapin	**-0.05**	-0.01	-0.04	-0.01	0.00	-0.01	-0.01	0.03
Glucobrassicanapin	-0.03	**0.04**	-0.02	-0.02	0.00	0.02	**0.06**	**0.06**
Hydroxyglucobrassicin	-0.01	-0.02	-0.03	**-0.07**	**-0.09**	-0.01	-0.03	-0.01
Glucobrassicin	-0.03	0.00	-0.02	0.01	-0.03	-0.01	-0.04	-0.01
Neoglucobrassicin	0.02	**-0.05**	-0.02	-0.02	**-0.07**	0.00	**-0.06**	-0.02
Gluconasturtiin	-0.03	-0.01	**-0.04**	0.03	0.00	0.03	-0.02	**0.05**
Methoxyglucobrassicin	0.01	-0.01	0.01	-0.02	-0.04	0.00	-0.08	0.01

Supplementary Table 2: **Effects of bee-pollination, aphid-herbivory, soil type and their interactions on absolute evolutionary rates for different traits categories: plant morphology, floral scent and leaf glucosinolates.** Evolutionary rates were assessed in haldanes (s.d. per generation). Significance was determined using a two-sided linear mixed model (LMM) with absolute evolutionary rates as dependent variable, pollination, herbivory, soil, trait groups and their interaction as fixed factors and replicate as random factors. Multiple-comparison post hoc tests were ran using estimated marginal means (EMMs) and their contrasts were computed with the emmeans package[1]. P-values are Tukey-corrected for multiple comparisons. Bold indicates significant factor effect (P<0.05) on trait evolutionary rate.

	N	Factors	df	χ^2	P
Evolutionary rates	528	**Pollination (P)**	**1**	**8.82**	**0.003**
		Herbivory (H)	1	0.54	0.464
		**Soil (S)**	**1**	**8.04**	**0.005**
		Trait groups (Tg)	2	0.30	0.862
		Replicate	1	0.10	0.748
		(P) x (H)	1	0.00	0.946
		(P) x (Tg)	2	0.40	0.818
		**(P) x (S)**	**1**	**4.56**	**0.033**
		(H) x (Tg)	2	0.69	0.708
		(H) x (S)	1	0.17	0.678
		(S) x (Tg)	2	4.90	0.086
		(P) x (H) x (S)	1	0.01	0.907
		(P) x (H) x (Tg)	2	2.99	0.224
		(P) x (S) x (Tg)	2	1.07	0.584
		(H) x (S) x (Tg)	2	2.39	0.303
		(P) x (H) x (S) x (Tg)	2	0.31	0.857

	Post hoc tests			
Parameters	Treatment groups	N	t-values	P
Limestone	Bee-pollination vs Hand-pollination	264	0.70	0.897
**Tuff**	**Bee-pollination vs Hand-pollination**	**264**	**3.67**	**0.002**

Supplementary Table 3: **Effects of bee-pollination, aphid-herbivory, soil type and their interactions on absolute evolutionary rates for different traits categories: plant morphology, floral scent and leaf glucosinolates.** Evolutionary rates were assessed in haldanes (s.d. per generation). Plants that evolved in different soils are analyzed separately to disentangle the role of herbivory and pollination in driving plant evolution in each soil type. Significance was determined using two-sided linear mixed models (LMM) with absolute evolutionary rates as dependent variable, pollination, herbivory, trait groups and their interaction as fixed factors and replicate as random factors. Significant P-values ($P<0.05$) are given in bold.

		Limestone				Tuff			
	Factors	N	df	χ^2	P	N	df	χ^2	P
Evolutionary rates	**Pollination (P)**	264	1	0.46	0.500	**264**	**1**	**10.66**	**0.001**
	Herbivory (H)		1	0.07	0.797		1	0.54	0.463
	Trait groups (Tg)		2	1.92	0.384		2	3.05	0.218
	Replicate		1	1.28	0.259		1	0.74	0.389
	(P) x (H)		1	0.00	0.969		1	0.01	0.906
	(P) x (Tg)		2	1.72	0.422		2	0.13	0.938
	(H) x (Tg)		2	1.47	0.480		2	1.60	0.449
	(P) x (H) x (Tg)		2	3.36	0.186		2	0.60	0.740

Supplementary Table 4: **Summary of post hoc analyses and multiple comparisons of linear discriminant analyses.** Significance was determined using two-sided linear mixed models (LMM) with linear discriminant scores as dependent variable, treatment as fixed factor and replicate as random factors. LH: limestone-line plants (L) growing with aphid-herbivory (H). LNH: limestone-line plants (T) growing without herbivory (NH) and bee-pollination (B). TH tuff-line plants (T) growing with aphid-herbivory (H). TNH: tuff-line plants (T) growing without herbivory (NH). P-values are Tukey-corrected for multiple comparisons. Bold indicates significant factor effects ($P<0.05$).

	Bee-pollination (N=283)		Hand-pollination (N=281)	
Parameters	t-ratio	P	t-ratio	P
G1 - LH	-0.17	0.215	0.25	0.078
G1 - LNH	**0.31**	**0.028**	**0.39**	**0.006**
G1 - TH	**1.14**	**$1.98^{*}10^{-14}$**	0.28	0.072
G1 - TNH	**1.20**	**$1.32^{*}10^{-17}$**	**0.81**	**$1.77^{*}10^{-08}$**
LH - LNH	**0.47**	**$7.76^{*}10^{-04}$**	0.14	0.379
LH - TH	**1.31**	**$1.85^{*}10^{-17}$**	0.02	0.860
LH - TNH	**1.37**	**$8.35^{*}10^{-21}$**	**0.56**	**$1.98^{*}10^{-04}$**
LNH - TH	**0.83**	**$3.29^{*}10^{-08}$**	-0.11	0.464
LNH - TNH	**0.89**	**$2.68^{*}10^{-10}$**	**0.42**	**0.004**
TH - TNH	0.06	0.658	**0.54**	**$5.20^{*}10^{-04}$**

Supplementary Table 5: Effects of experimental treatments on plant trait evolution. Significance was determined using two-sided linear mixed models (LMM) with individual trait as dependent variable, treatment and its interactions with replicate as fixed factors, and replicate as random factors. Bold indicates significant factor effects (P<0.05).

	Treatment				Replicate			Treatment x Replicate		
	N	df	χ^2	P	df	χ^2	P	df	χ^2	P
Morphology										
Height day 20 (cm)	575	7	**94.26**	**$1.65^{*}10^{-17}$**	**1**	61.03	0.708	7	**61.03**	**$9.40^{*}10^{-11}$**
Height day 30 (cm)	575	7	**92.20**	**$4.38^{*}10^{-17}$**	**1**	0.15	0.697	7	**18.17**	**0.011**
Leaf size (cm^2)	575	7	**124.31**	**$9.70^{*}10^{-24}$**	**1**	0.00	0.997	7	**26.67**	**$3.81^{*}10^{-04}$**
Time to flower (day)	575	7	**89.25**	**$1.76^{*}10^{-16}$**	**1**	0.05	0.832	7	**43.74**	**$2.40^{*}10^{-07}$**
Cumulative branches length (cm)	575	7	**18.79**	**0.009**	**1**	0.02	0.877	7	**41.96**	**$5.30^{*}10^{-05}$**
Number of flowering branches	575	7	**14.05**	**0.050**	**1**	0.02	0.877	7	**46.52**	**$6.92^{*}10^{-08}$**
Flower production rate (per day)	575	7	**33.85**	**$1.99^{*}10^{-05}$**	**1**	0.25	0.620	7	9.83	0.277
Flower number	575	7	**49.28**	**$2.00^{*}10^{-08}$**	**1**	0.02	0.902	7	11.94	0.102
Nectar amount (nL/flower)	575	7	**41.95**	**$5.33^{*}10^{-07}$**	**1**	0.02	0.875	7	**27.62**	**$2.58^{*}10^{-04}$**
Flower diameter (mm)	575	7	**51.94**	**$6.00^{*}10^{-09}$**	**1**	0.42	0.517	7	13.97	0.052
Petal length (cm)	564	7	**37.71**	**$3.44^{*}10^{-08}$**	**1**	1.37	0.241	7	**17.61**	**0.014**
Petal width (cm)	564	7	**58.61**	**$2.85^{*}10^{-10}$**	**1**	0.51	0.474	7	**39.52**	**$1.56^{*}10^{-06}$**
Sepal length (cm)	564	7	**22.91**	**0.002**	**1**	0.20	0.656	7	**31.34**	**$5.38^{*}10^{-05}$**
Style length (cm)	564	7	11.27	0.127	1	1.35	0.245	7	**26.49**	**$4.11^{*}10^{-04}$**
Stamen length (cm)	564	7	**14.58**	**0.042**	**1**	3.81	0.051	7	**21.07**	**0.004**
Herkogamy (cm)	564	7	9.05	0.249	1	0.50	0.479	7	13.82	0.055
Bioassays										
Number of aphid visits	568	7	7.58	0.372	1	0.39	0.532	7	8.48	0.293
Number of bee visits	568	7	**32.21**	**$3.71^{*}10^{-05}$**	**1**	0.08	0.781	7	11.94	0.102
Floral scent(pg.l^{-1}.hr^{-1}.$flower^{-1}$)										
**Aromatic compounds**										
Benzaldehyde	564	7	**56.08**	**$9.35^{*}10^{-07}$**	**1**	0.00	0.993	7	**15.75**	**0.027**
Methyl benzoate	564	7	**52.56**	**$2.52^{*}10^{-08}$**	**1**	0.19	0.662	7	**17.91**	**$1.34^{*}10^{-05}$**
Phenylethyl alcohol	564	7	**42.58**	**$2.34^{*}10^{-04}$**	**1**	0.94	0.331	7	**25.00**	**$7.58^{*}10^{-04}$**
Phenylacetaldehyde	564	7	**82.03**	**$3.39^{*}10^{-14}$**	**1**	0.64	0.423	7	**32.73**	**$2.97^{*}10^{-05}$**
p-Anisaldehyde	564	7	**17.55**	**0.014**	**1**	0.05	0.815	7	**29.07**	**$1.41^{*}10^{-04}$**
2-Aminobenzaldehyde	564	7	**19.75**	**0.006**	**1**	0.03	0.854	7	**26.95**	**$3.40^{*}10^{-04}$**
Methyl salicylate	564	7	**25.31**	**$6.69^{*}10^{-04}$**	**1**	0.00	0.999	7	**28.93**	**$1.49^{*}10^{-04}$**
**Terpenoids**										
β-Pinene	564	7	2.43	0.932	**1**	0.17	0.681	7	3.77	0.806
(E,E)-α-Farnesene	564	7	**34.97**	**$1.13^{*}10^{-05}$**	**1**	0.45	0.505	7	13.31	0.065
**Fatty acid derivatives**										
(Z)-3-Hexen-1-ol-acetate	564	7	**33.38**	**$2.24^{*}10^{-05}$**	**1**	0.00	0.958	7	12.88	0.075

**Nitrogen containing compounds**										
Methyl anthranilate	564	7	**120.66**	**$5.59^{*}10^{-23}$**	**1**	0.11	0.745	7	**57.24**	**$5.34^{*}10^{-10}$**
Benzyl nitrile	564	7	**17.31**	**0.016**	**1**	0.17	0.683	7	**17.86**	**0.013**
Indole	564	7	13.03	0.071	1	0.08	0.772	7	**28.85**	**$1.54^{*}10^{-04}$**
**Sulphur containing compounds**										
1-Butene-4-isothiocyanate	564	7	**16.57**	**0.020**	**1**	0.26	0.607	7	7.73	0.357
**Total emission**	564	7	**28.08**	**$1.98^{*}10^{-04}$**	1	0.00	0.998	7	13.19	0.067
Leaf glucosinolates (pg.mg^{-1})										
Glucoraphin	485	7	**22.79**	**0.002**	1	0.06	0.801	7	10.38	0.168
Glucoalyssin	500	7	**19.71**	**0.006**	1	0.42	0.517	7	**22.35**	**0.002**
Sinalbin	512	7	3.67	0.817	1	0.01	0.903	7	8.45	0.294
Gluconapin	512	7	**20.39**	**0.005**	1	0.03	0.872	7	12.00	0.100
Glucobrassicanapin	509	7	**58.74**	**$3.16^{*}10^{-11}$**	1	0.02	0.883	7	**22.96**	**0.002**
Hydroxyglucobrassicin	510	7	**31.47**	**$7.77^{*}10^{-04}$**	1	0.05	0.821	7	**28.68**	**0.001**
Glucobrassicin	510	7	**14.43**	**0.044**	1	0.11	0.738	7	**18.71**	**0.009**
Neoglucobrassicin	511	7	**36.45**	**$4.88^{*}10^{-06}$**	1	0.09	0.766	7	**22.55**	**0.002**
Gluconasturtiin	512	7	**49.38**	**$1.91^{*}10^{-08}$**	1	1.85	0.174	7	**55.06**	**$1.45^{*}10^{-09}$**
Methoxyglucobrassicin	511	7	**27.11**	**$4.17^{*}10^{-04}$**	1	0.22	0.640	7	**19.12**	**0.008**
**Total glucosinolates**	477	7	**18.90**	**0.009**	1	0.07	0.794	7	**17.71**	**0.040**

Supplementary Table 6: Effects of pollination (bee-pollination or hand-pollination), herbivory (aphid-herbivory or no herbivory), and soil (limestone or tuff soil), and their interactions on plant-trait evolution estimated by two-sided linear mixed models (LMM). Bold indicates significant factors effects (P<0.05) in driving divergence between treatments.

Traits	N	Factor	df	χ^2	P	sign
Morphology						
Height day 20 (cm)	575	**Pollination (P)**	**1**	**20.87**	**$4.92^{*}10^{-06}$**	-
		Herbivory (H)	1	0.00	0.966	
		**Soil (S)**	**1**	**18.79**	**$1.46^{*}10^{-05}$**	-
		Replicate	1	0.13	0.720	
		(S) x (H)	1	3.53	0.060	
		**(S) x (P)**	**1**	**36.23**	**$1.75^{*}10^{-09}$**	
		(P) x (H)	1	0.67	0.413	
		**(S) x (P) x (H)**	**1**	**6.01**	**0.014**	
Height day 30 (cm)	575	Pollination (P)	1	2.40	0.122	
		**Herbivory (H)**	**1**	**6.31**	**0.012**	-
		**Soil (S)**	**1**	**30.89**	**$2.73^{*}10^{-08}$**	+
		Replicate	1	0.15	0.699	
		**(S) x (H)**	**1**	**19.58**	**$9.63^{*}10^{-06}$**	
		**(S) x (P)**	**1**	**19.26**	**$1.14^{*}10^{-05}$**	
		**(P) x (H)**	**1**	**12.02**	**$5.27^{*}10^{-04}$**	
		(S) x (P) x (H)	1	0.06	0.814	
Time to flower (day)	575	**Pollination (P)**	**1**	**7.94**	**0.005**	+
		Herbivory (H)	1	0.00	0.991	
		**Soil (S)**	**1**	**25.77**	**$3.85^{*}10^{-07}$**	+
		Replicate	1	0.04	0.837	
		(S) x (H)	1	0.70	0.404	
		**(S) x (P)**	**1**	**48.57**	**$3.18^{*}10^{-12}$**	
		(P) x (H)	1	0.15	0.702	

		(S) x (P) x (H)	1	0.72	0.395	
Leaf size (cm^2)	575	**Pollination (P)**	**1**	**11.97**	**$5.42^{*}10^{-04}$**	+
		**Herbivory (H)**	**1**	**6.45**	**0.011**	-
		**Soil (S)**	**1**	**54.43**	**$1.61^{*}10^{-13}$**	+
		Replicate	1	0.00	0.997	
		(S) x (H)	1	3.69	0.055	
		**(S) x (P)**	**1**	**39.82**	**$2.79^{*}10^{-10}$**	
		(P) x (H)	1	2.92	0.088	
		(S) x (P) x (H)	1	1.01	0.315	
Cumulative branches length (cm)	575	Pollination (P)	1	0.54	0.463	
		Herbivory (H)	1	0.40	0.527	
		Soil (S)	1	0.10	0.750	
		Replicate	1	0.02	0.880	
		**(S) x (H)**	**1**	**6.38**	**0.012**	
		(S) x (P)	1	1.30	0.253	
		**(P) x (H)**	**1**	**8.70**	**0.003**	
		(S) x (P) x (H)	1	0.28	0.596	
Number of flowering branches	575	Pollination (P)	1	0.03	0.862	
		Herbivory (H)	1	1.40	0.236	
		Soil (S)	1	1.04	0.308	
		Replicate	1	0.02	0.881	
		(S) x (H)	1	2.20	0.138	
		**(S) x (P)**	**1**	**5.83**	**0.016**	
		(P) x (H)	1	0.52	0.470	
		(S) x (P) x (H)	1	2.09	0.149	
Flower production rate (per day)	575	Pollination (P)	1	0.96	0.327	
		Herbivory (H)	1	1.16	0.280	
		**Soil (S)**	**1**	**7.72**	**0.005**	+
		Replicate	1	0.38	0.537	
		**(S) x (H)**	**1**	**12.35**	**$1.11^{*}10^{-04}$**	
		**(S) x (P)**	**1**	**4.74**	**0.029**	
		(P) x (H)	1	0.73	0.392	
		(S) x (P) x (H)	**1**	2.99	0.084	
Number of flowers	575	Pollination (P)	1	0.12	0.733	
		Herbivory (H)	1	1.71	0.191	
		**Soil (S)**	**1**	**9.53**	**0.002**	+
		Replicate	1	0.02	0.902	
		**(S) x (H)**	**1**	**10.47**	**0.001**	
		**(S) x (P)**	**1**	**15.57**	**$7.95^{*}10^{-05}$**	
		(P) x (H)	1	0.06	0.802	
		**(S) x (P) x (H)**	**1**	**11.45**	**$7.17^{*}10^{-04}$**	
Nectar amount (nL/flower)	563	Pollination (P)	1	0.45	0.504	
		Herbivory (H)	1	2.79	0.095	
		**Soil (S)**	**1**	**29.11**	**$6.85^{*}10^{-08}$**	+
		Replicate	1	0.02	0.878	
		(S) x (H)	1	0.71	0.399	
		**(S) x (P)**	**1**	**70.03**	**0.008**	
		(P) x (H)	1	0.15	0.697	
		(S) x (P) x (H)	1	0.48	0.488	
Flower diameter (cm)	564	**Pollination (P)**	**1**	**44.63**	**$2.38^{*}10^{-05}$**	+

		Herbivory (H)	1	0.85	0.355	
		Soil (S)	1	0.39	0.533	
		Replicate	1	0.12	0.734	
		(S) x (H)	1	2.52	0.113	
		(S) x (P)	1	0.76	0.383	
		(P) x (H)	1	0.72	0.396	
		(S) x (P) x (H)	1	1.52	0.218	
Petal length (cm)	564	**Pollination (P)**	**1**	**29.10**	**$6.86^{*}10^{-08}$**	+
		Herbivory (H)	1	0.00	0.982	
		Soil (S)	1	0.91	0.340	
		Replicate	1	1.35	0.246	
		(S) x (H)	1	1.62	0.203	
		**(S) x (P)**	**1**	**3.84**	**0.050**	
		(P) x (H)	1	0.04	0.832	
		(S) x (P) x (H)	1	1.46	0.228	
Petal width (cm)	564	**Pollination (P)**	**1**	**30.40**	**$3.52^{*}10^{-08}$**	+
		**Herbivory (H)**	**1**	**16.98**	**$3.78^{*}10^{-05}$**	-
		Soil (S)	1	0.62	0.431	
		Replicate	1	0.48	0.487	
		(S) x (H)	1	3.70	0.055	
		(S) x (P)	1	2.85	0.091	
		(P) x (H)	1	1.30	0.254	
		(S) x (P) x (H)	1	0.03	0.854	
Sepal length (cm)	564	Pollination (P)	1	0.84	0.359	
		**Herbivory (H)**	**1**	**5.45**	**0.020**	-
		Soil (S)	1	1.18	0.278	
		Replicate	1	0.19	0.663	
		**(S) x (H)**	**1**	**9.86**	**0.002**	
		(S) x (P)	1	1.42	0.233	
		(P) x (H)	1	2.37	0.124	
		(S) x (P) x (H)	1	0.79	0.375	
Style length (cm)	564	Pollination (P)	1	0.09	0.764	
		Herbivory (H)	1	2.80	0.094	
		Soil (S)	1	0.42	0.518	
		Replicate	1	1.30	0.254	
		(S) x (H)	1	1.68	0.195	
		(S) x (P)	1	1.03	0.311	
		(P) x (H)	1	3.00	0.083	
		(S) x (P) x (H)	1	1.86	0.173	
Stamen length (cm)	564	Pollination (P)	1	0.00	0.978	
		Herbivory (H)	1	1.54	0.139	
		**Soil (S)**	**1**	**5.09**	**0.024**	+
		Replicate	1	3.71	0.054	
		(S) x (H)	1	0.14	0.709	
		(S) x (P)	1	0.26	0.613	
		**(P) x (H)**	**1**	**4.64**	**0.031**	
		(S) x (P) x (H)	1	2.60	0.107	
Herkogamy (cm)	564	Pollination (P)	1	0.00	0.947	
		Herbivory (H)	1	2.53	0.112	
		Soil (S)	1	0.19	0.667	
		Replicate	1	0.49	0.482	

		(S) x (H)	1	0.92	0.337	
		(S) x (P)	1	0.42	0.516	
		**(P) x (H)**	**1**	**4.14**	**0.042**	
		(S) x (P) x (H)	1	0.74	0.389	
Bioassays						
Number of aphid visits	575	Pollination (P)	1	2.81	0.094	
		Herbivory (H)	1	0.10	0.750	
		Soil (S)	1	1.71	0.191	
		Replicate	1	0.39	0.533	
		(S) x (H)	1	0.69	0.405	
		(S) x (P)	1	0.27	0.603	
		(P) x (H)	1	1.98	0.160	
		(S) x (P) x (H)	1	0.00	0.960	
Number of bee visits	575	**Pollination (P)**	**1**	**8.27**	**0.004**	+
		Herbivory (H)	1	1.44	0.230	
		**Soil (S)**	**1**	**5.13**	**0.023**	+
		Replicate	1	0.08	0.782	
		**(S) x (H)**	**1**	**5.36**	**0.021**	
		(S) x (P)	1	2.41	0.120	
		**(P) x (H)**	**1**	**8.56**	**0.003**	
		(S) x (P) x (H)	1	0.79	0.375	
Floral scent ($pg.l^{-1}.hr^{-1}.flower^{-1}$)						
**Aromatic compounds**						
Benzaldehyde	564	Pollination (P)	1	0.03	0.859	
		Herbivory (H)	1	1.73	0.188	
		**Soil (S)**	**1**	**22.19**	**$2.47^{*}10^{-06}$**	+
		Replicate	1	0.00	0.993	
		**(S) x (H)**	**1**	**20.59**	**$5.69^{*}10^{-06}$**	
		(S) x (P)	1	0.09	0.761	
		(P) x (H)	1	0.43	0.512	
		**(S) x (P) x (H)**	**1**	**10.09**	**0.001**	
Methyl benzoate	564	**Pollination (P)**	**1**	**5.17**	**0.023**	+
		Herbivory (H)	1	1.69	0.193	
		**Soil (S)**	**1**	**16.94**	**$3.86^{*}10^{-05}$**	-
		Replicate	1	0.19	0.665	
		(S) x (H)	1	1.13	0.287	
		(S) x (P)	1	0.81	0.368	
		(P) x (H)	1	0.32	0.573	
		**(S) x (P) x (H)**	**1**	**25.54**	**$4.39^{*}10^{-07}$**	
Phenylethyl alcohol	564	**Pollination (P)**	**1**	**4.73**	**0.030**	-
		**Herbivory (H)**	**1**	**8.02**	**0.005**	-
		**Soil (S)**	**1**	**24.20**	**$8.68^{*}10^{-07}$**	-
		Replicate	1	0.53	0.469	
		(S) x (H)	1	2.08	0.150	
		(S) x (P)	1	0.02	0.882	
		(P) x (H)	1	0.48	0.489	
		(S) x (P) x (H)	1	1.71	0.191	
Phenylacetaldehyde	564	**Pollination (P)**	**1**	**6.67**	**0.010**	-
		**Herbivory (H)**	**1**	**8.56**	**0.003**	-
		**Soil (S)**	**1**	**47.12**	**$6.66^{*}10^{-12}$**	-
		Replicate	1	0.61	0.433	

		(S) x (H)	1	3.40	0.065	
		(S) x (P)	1	0.06	0.799	
		(P) x (H)	1	0.82	0.365	
		**(S) x (P) x (H)**	**1**	**11.71**	**$6.21^{*}10^{-04}$**	
p-Anisaldehyde	564	Pollination (P)	1	1.26	0.261	
		Herbivory (H)	1	0.43	0.510	
		Soil (S)	1	0.04	0.843	
		Replicate	1	0.05	0.819	
		**(S) x (H)**	**1**	**11.39**	**$7.38^{*}10^{-04}$**	
		(S) x (P)	1	0.27	0.602	
		(P) x (H)	1	1.20	0.274	
		(S) x (P) x (H)	1	2.37	0.123	
2-Aminobenzaldehyde	564	Pollination (P)	1	2.23	0.135	
		Herbivory (H)	1	1.38	0.240	
		**Soil (S)**	**1**	**3.88**	**0.049**	-
		Replicate	1	0.30	0.856	
		**(S) x (H)**	**1**	**4.04**	**0.045**	
		**(S) x (P)**	**1**	**4.38**	**0.036**	
		(P) x (H)	1	0.69	0.406	
		(S) x (P) x (H)	1	2.49	0.115	
Methyl salicylate	564	Pollination (P)	1	3.29	0.070	
		Herbivory (H)	1	0.69	0.408	
		Soil (S)	1	3.59	0.058	
		Replicate	1	0.00	0.999	
		(S) x (H)	1	0.98	0.322	
		**(S) x (P)**	**1**	**6.77**	**0.009**	
		(P) x (H)	1	0.89	0.346	
		**(S) x (P) x (H)**	**1**	**8.10**	**0.004**	
**Terpenoïds**						
β-Pinene	564	Pollination (P)	1	0.39	0.534	
		Herbivory (H)	1	0.64	0.425	
		Soil (S)	1	0.01	0.930	
		Replicate	1	0.17	0.680	
		(S) x (H)	1	0.43	0.510	
		(S) x (P)	1	0.15	0.701	
		(P) x (H)	1	0.04	0.848	
		(S) x (P) x (H)	1	0.77	0.379	
(E,E)-α-Farnesene	564	Pollination (P)	1	3.46	0.063	
		Herbivory (H)	1	2.29	0.130	
		**Soil (S)**	**1**	**4.75**	**0.029**	+
		Replicate	1	0.44	0.507	
		**(S) x (H)**	**1**	**10.68**	**0.001**	
		**(S) x (P)**	**1**	**6.57**	**0.010**	
		**(P) x (H)**	**1**	**4.50**	**0.034**	
		(S) x (P) x (H)	1	2.35	0.125	
**Fatty acid derivatives**						
(Z)-3-Hexen-1-ol-acetate	564	Pollination (P)	1	0.02	0.898	
		**Herbivory (H)**	**1**	**3.99**	**0.046**	-
		Soil (S)	1	1.29	0.256	
		Replicate	1	0.00	0.958	
		(S) x (H)	1	1.31	0.252	
		**(S) x (P)**	**1**	**6.46**	**0.011**	

		**(P) x (H)**	**1**	**6.24**	**0.013**	
		**(S) x (P) x (H)**	**1**	**13.88**	**$1.95^{*}10^{-04}$**	
**Nitrogen containing compounds**						
Methyl anthranilate	564	Pollination (P)	1	0.60	0.438	
		**Herbivory (H)**	**1**	**16.12**	**$5.94^{*}10^{-05}$**	+
		**Soil (S)**	**1**	**48.18**	**$3.88^{*}10^{-12}$**	-
		Replicate	1	0.10	0.755	
		**(S) x (H)**	**1**	**17.06**	**$3.63^{*}10^{-05}$**	
		**(S) x (P)**	**1**	**5.40**	**0.020**	
		(P) x (H)	1	0.07	0.786	
		**(S) x (P) x (H)**	**1**	**23.17**	**$1.48^{*}10^{-06}$**	
Benzyl nitrile	564	**Pollination (P)**	**1**	**3.88**	**0.049**	+
		Herbivory (H)	1	1.24	0.265	
		**Soil (S)**	**1**	**18.36**	**$1.83^{*}10^{-05}$**	-
		Replicate	1	0.25	0.620	
		(S) x (H)	1	0.01	0.917	
		(S) x (P)	1	2.06	0.152	
		(P) x (H)	1	0.58	0.447	
		(S) x (P) x (H)	1	2.10	0.147	
Indole	564	Pollination (P)	1	0.85	0.356	
		**Herbivory (H)**	**1**	**4.16**	**0.042**	+
		Soil (S)	1	2.16	0.142	
		Replicate	1	0.08	0.776	
		(S) x (H)	1	0.28	0.595	
		(S) x (P)	1	2.90	0.089	
		(P) x (H)	1	0.41	0.524	
		(S) x (P) x (H)	1	1.82	0.177	
**Total N-containing volatile compounds**	564	Pollination (P)	1	2.08	0.149	
		Herbivory (H)	1	3.64	0.057	
		**Soil (S)**	**1**	**9.75**	**0.002**	-
		Replicate	1	0.01	0.934	
		(S) x (H)	1	0.34	0.559	
		(S) x (P)	1	1.11	0.291	
		(P) x (H)	1	0.24	0.627	
		(S) x (P) x (H)	1	3.50	0.061	
**Sulfphur containing compounds**						
1-Butene-4-isothiocyanate	564	Pollination (P)	1	3.22	0.073	
		**Herbivory (H)**	**1**	**7.76**	**0.005**	-
		Soil (S)	1	0.15	0.694	
		Replicate	1	0.26	0.607	
		(S) x (H)	1	3.76	0.053	
		(S) x (P)	1	0.02	0.896	
		(P) x (H)	1	0.01	0.905	
		(S) x (P) x (H)	1	1.65	0.199	
**Total emission**	564	Pollination (P)	1	0.10	0.756	
		Herbivory (H)	1	0.36	0.547	
		**Soil (S)**	**1**	**8.89**	**0.003**	-
		Replicate	1	0.00	0.992	
		**(S) x (H)**	**1**	**4.12**	**0.042**	
		(S) x (P)	1	1.18	0.278	
		(P) x (H)	1	0.03	0.860	
		**(S) x (P) x (H)**	**1**	**14.07**	**$1.51^{*}10^{-04}$**	

Leaf glucosinolates (pg.mg^{-1})						
Glucoraphanin	485	**Pollination (P)**	**1**	**13.04**	**$3.05^{*}10^{-04}$**	-
		Herbivory (H)	1	2.09	0.149	
		Soil (S)	1	0.51	0.475	
		Replicate	1	0.06	0.802	
		(S) x (H)	1	1.44	0.230	
		(S) x (P)	1	0.46	0.496	
		(P) x (H)	1	0.05	0.820	
		**(S) x (P) x (H)**	**1**	**4.81**	**0.028**	
Glucoalyssin	500	Pollination (P)	1	0.09	0.760	
		**Herbivory (H)**	**1**	**4.76**	**0.029**	-
		Soil (S)	1	0.05	0.830	
		Replicate	1	0.41	0.523	
		**(S) x (H)**	**1**	**10.91**	**$9.58^{*}10^{-04}$**	
		(S) x (P)	1	0.14	0.712	
		(P) x (H)	1	0.39	0.531	
		(S) x (P) x (H)	1	2.81	0.094	
Sinalbin	512	Pollination (P)	1	0.50	0.481	
		Herbivory (H)	1	0.29	0.591	
		Soil (S)	1	2.52	0.112	
		Replicate	1	0.01	0.904	
		(S) x (H)	1	0.04	0.836	
		(S) x (P)	1	0.00	0.996	
		(P) x (H)	1	0.31	0.579	
		(S) x (P) x (H)	1	0.00	0.946	
Gluconapin	512	**Pollination (P)**	**1**	**5.05**	**0.025**	-
		Herbivory (H)	1	1.30	0.254	
		**Soil (S)**	**1**	**8.69**	**0.003**	+
		Replicate	1	0.03	0.873	
		(S) x (H)	1	0.17	0.683	
		(S) x (P)	1	1.84	0.175	
		(P) x (H)	1	1.00	0.316	
		(S) x (P) x (H)	1	1.98	0.159	
Glucobrassicanapin	509	**Pollination (P)**	**1**	**6.74**	**0.009**	-
		Herbivory (H)	1	2.21	0.137	
		**Soil (S)**	**1**	**19.83**	**$8.47^{*}10^{-06}$**	+
		Replicate	1	0.02	0.885	
		**(S) x (H)**	**1**	**20.18**	**$7.04^{*}10^{-06}$**	
		(S) x (P)	1	1.99	0.158	
		(P) x (H)	1	2.74	0.098	
		(S) x (P) x (H)	1	3.25	0.072	
Hydroxyglucobrassicin	510	Pollination (P)	1	2.63	0.105	
		Herbivory (H)	1	0.20	0.655	
		Soil (S)	1	0.72	0.397	
		Replicate	1	0.05	0.825	
		**(S) x (H)**	**1**	**9.07**	**0.003**	
		**(S) x (P)**	**1**	**13.36**	**$2.67^{*}10^{-04}$**	
		(P) x (H)	1	3.73	0.054	
		(S) x (P) x (H)	1	0.27	0.604	
Glucobrassicin	510	**Pollination (P)**	**1**	**11.05**	**0.002**	-
		Herbivory (H)	1	0.19	0.650	

		Soil (S)	1	3.48	0.080	
		Replicate	1	0.13	0.741	
		(S) x (H)	1	0.43	0.489	
		(S) x (P)	1	0.02	0.859	
		(P) x (H)	1	0.25	0.621	
		(S) x (P) x (H)	1	0.00	0.945	
Neoglucobrassicin	511	Pollination (P)	1	1.58	0.223	
		Herbivory (H)	1	0.96	0.346	
		**Soil (S)**	**1**	**4.13**	**0.048**	-
		Replicate	1	0.08	0.770	
		(S) x (H)	1	0.03	0.852	
		**(S) x (P)**	**1**	**21.21**	**$1.06^{*}10^{-05}$**	
		(P) x (H)	1	2.01	0.166	
		**(S) x (P) x (H)**	**1**	**7.70**	**0.005**	
Gluconasturtiin	512	**Pollination (P)**	**1**	**33.52**	**$7.05^{*}10^{-09}$**	-
		Herbivory (H)	1	0.13	0.718	
		**Soil (S)**	**1**	**5.41**	**0.020**	+
		Replicate	1	1.69	0.194	
		(S) x (H)	1	0.00	0.947	
		(S) x (P)	1	0.52	0.469	
		**(P) x (H)**	**1**	**4.74**	**0.030**	
		(S) x (P) x (H)	1	0.53	0.465	
Methoxyglucobrassicin	511	Pollination (P)	1	2.20	0.150	
		Herbivory (H)	1	0.32	0.579	
		**Soil (S)**	**1**	**6.30**	**0.013**	-
		Replicate	1	0.21	0.644	
		(S) x (H)	1	0.04	0.827	
		**(S) x (P)**	**1**	**16.12**	**$7.03^{*}10^{-05}$**	
		(P) x (H)	1	1.19	0.277	
		(S) x (P) x (H)	1	1.16	0.340	
**Total glucosinolates**	477	**Pollination (P)**	**1**	**4.69**	**0.030**	-
		Herbivory (H)	1	2.72	0.099	
		**Soil (S)**	**1**	**6.62**	**0.010**	+
		Replicate	1	0.07	0.796	
		(S) x (H)	1	0.23	0.634	
		(S) x (P)	1	1.32	0.251	
		(P) x (H)	1	0.56	0.453	
		(S) x (P) x (H)	1	2.16	0.141	

Supplementary Table 7: Trait differences (mean ± SD) among first generation plants and those having evolved with or without aphid-herbivory, with or without bee-pollination and either in limestone or tuff soil. Significance between treatments was determined using two-sided linear mixed models (LMM) with individual traits as dependent variable, treatment and its interactions with replicate as fixed factors, and replicate as random factors. Different letters below each treatment group mean indicate significant differences between treatment groups (P<0.05); P-values are Tukey-corrected for multiple comparisons. G1: plants of generation one. LHB: limestone line plants (L) growing with aphid-herbivory (H) and bee-pollination (B). LHH: limestone line plants (L) growing with aphid-herbivory (H) and hand-pollination (H). LNHB: limestone line plants (L) growing without herbivory (NH) and bee-pollination (B). LNHH: limestone line plants (L) growing without herbivory (NH) and hand-pollination (H). THB: tuff line plants (T) growing with aphid-herbivory (H) and bee-pollination (B). THH: tuff line plants (T) growing with aphid-herbivory (H) and hand-pollination (H). TNHB: tuff line plants (T) growing without herbivory (NH) and bee-pollination (B). TNHH: tuff line plants (T) growing without herbivory (NH) and hand-pollination (H).

Traits	N	G1	LHB	LHH	LNHB	LNHH	THB	THH	TNHB	TNHH
Morphology										
Height day 20 (cm)	647	24.70 ± 8.02	24.77 ± 7.06	22.74 ± 8.18	22.44 ± 6.46	22.55 ± 8.12	15.16 ± 7.65	24.23 ± 9.70	18.50 ± 7.53	23.29 ± 7.70
			A	A	A	A	B	A	B	A
							**$1.99^{*}10^{-11}$**		**$4.43^{*}10^{-06}$**	
Height day 30 (cm)	647	48.05 ± 8.03	42.84 ± 8.24	49.07 ± 8.87	43.73 ± 7.96	45.57 ± 9.00	46.42 ± 7.71	47.02 ± 8.47	53.64 ± 7.26	49.20 ± 7.49
			D	B	CD	BCD	BCD	BC	A	B
			**$1.81^{*}10^{-04}$**		**0.001**				**$2.33^{*}10^{-05}$**	
Leaf size (cm^2)	647	40.11 ± 2.22	35.69 ± 1.74	43.25 ± 1.95	40.19 ± 1.58	40.40 ± 1.52	52.97 ± 2.00	40.70 ± 2.48	59.96 ± 2.07	45.79 ± 1.81
			D	CD	CD	CD	AB	CD	A	BC
							**$3.09^{*}10^{-05}$**		**$9.67^{*}10^{-10}$**	**0.049**

Time to flower (day)	647	18.64 ± 1.92	18.69 ± 1.97	19.48 ± 1.84	18.92 ± 1.56	19.54 ± 1.94	21.01 ± 2.61	19.15 ± 2.27	20.67 ± 2.12	19.22 ± 1.70
			B	B	B	B	A	B	A	B
				**0.008**		**0.005**	**$6.31^{*}10^{-09}$**		**$1.39^{*}10^{-08}$**	
Cumulative branches length (cm)	647	90.21 ± 46.99	84.37 ± 40.25	94.93 ± 46.86	85.44 ± 42.22	72.13 ± 40.00	72.52 ± 36.45	87.25 ± 45.63	87.27 ± 36.69	85.41 ± 39.08
			AB	A	AB	B	B	AB	AB	AB
						**0.014**	**0.013**			
Number of flowering branches	647	4.89 ± 2.29	5.17 ± 1.93	5.15 ± 1.99	5.08 ± 1.80	4.36 ± 1.78	4.60 ± 2.00	4.90 ± 2.46	4.53 ± 1.65	5.07 ± 2.00
			A	A	A	A	A	A	A	A
Flower production rate (per day)	647	5.44 ± 0.22	5.27 ± 0.27	6.33 ± 0.28	5.30 ± 1.84	5.40 ± 1.80	5.86 ± 1.88	5.47 ± 1.85	6.55 ± 3.00	6.49 ± 2.42
			C	ABC	C	BC	ABC	ABC	A	AB
				**$1.98^{*}10^{-05}$**					**$1.11^{*}10^{-08}$**	**$3.48^{*}10^{-05}$**
Flower number	647	27.42 ± 14.90	27.71 ± 15.18	37.80 ± 16.62	29.86 ± 13.06	30.71 ± 14.24	36.81 ± 16.16	28.26 ± 14.78	38.60 ± 14.58	38.00 ± 16.76
			D	AB	CD	BCD	ABC	D	A	AB
				**$1.32^{*}10^{-04}$**			**$4.03^{*}10^{-04}$**		**$1.14^{*}10^{-05}$**	**$1.00^{*}10^{-04}$**

Nectar amount (nL/flower)	634	5.91 ± 3.50	5.40 ± 3.58	7.12 ± 4.33	6.14 ± 4.76	7.02 ± 5.13	8.47 ± 1.62	7.58 ± 4.46	9.33 ± 5.01	8.68 ± 3.97
			D	ABCD	CD	BCD	ABC	ABCD	A	AB
							**$8.72^{*}10^{-04}$**	**0.016**	**$5.87^{*}10^{-06}$**	**$2.13^{*}10^{-05}$**
Flower diameter (cm)	636	1,46 ± 0.14	1.48 ± 0.16	1.40 ± 0.15	1.51 ± 0.17	1.41 ± 0.12	1.51 ± 0.15	1.44 ± 0.18	1.53 ± 0.15	1.40 ± 0.14
			AB	C	AB	BC	AB	BC	A	C
				**0.035**	**0.038**		**0.035**		**0.006**	**0.024**
Petal length (cm)	636	1.06 ± 0.10	1.07 ± 0.10	1.03 ± 0.09	1.07 ± 0.08	1.05 ± 0.09	1.09 ± 0.10	1.04 ± 0.11	1.09 ± 0.11	1.02 ± 0.09
			AB	B	AB	AB	A	B	A	B
							**0.042**			**0.020**
Petal width (cm)	636	0.51 ± 0.07	0.53 ± 0.06	0.48 ± 0.07	0.55 ± 0.08	0.52 ± 0.06	0.52 ± 0.06	0.49 ± 0.07	0.53 ± 0.08	0.51 ± 0.07
			ABC	D	A	ABC	ABC	CD	AB	BC
				**0.001**	**0.004**					
Sepal length (cm)	636	0.55 ± 0.07	0.52 ± 0.07	0.53 ± 0.07	0.56 ± 0.07	0.56 ± 0.06	0.55 ± 0.08	0.55 ± 0.06	0.56 ± 0.09	0.53 ± 0.06
			B	AB	A	AB	AB	AB	A	AB
			**0.009**	**0.034**						

Style length (cm)	636	0.76 ± 0.10	0.73 ± 0.10	0.74 ± 0.13	0.73 ± 0.09	0.73 ± 0.11	0.75 ± 0.14	0.76 ± 0.12	0.75 ± 0.10	0.71 ± 0.09
			AB	AB	AB	AB	AB	A	AB	B
										**0.002**
Stamen length (cm)	636	0.67 ± 0.06	0.66 ± 0.07	0.66 ± 0.07	0.65 ± 0.06	0.66 ± 0.06	0.69 ± 0.07	0.67 ± 0.08	0.66 ± 0.08	0.68 ± 0.07
			AB	AB	B	AB	A	AB	AB	AB
Herkogamy (cm)	636	0.12 ± 0.06	0.12 ± 0.07	0.13 ± 0.08	0.12 ± 0.06	0.12 ± 0.06	0.12 ± 5.25	0.14 ± 0.08	0.12 ± 0.06	0.10 ± 0.06
			A	A	A	A	A	A	A	A
Bioassays										
Number of aphid visits	639	1.15 ± 1.74	1.00 ± 1.78	1.04 ± 1.61	1.13 ± 1.58	0.78 ± 1.02	1.14 ± 1.51	1.03 ± 1.26	1.47 ± 2.23	1.00 ± 1.51
			A	A	A	A	A	A	A	A
Number of bee visits	639	13	22	24	25	14	24	22	48	21
			B	B	B	B	B	B	A	B
									**$3.44^{*}10^{-06}$**	

Floral scent (pg.l^{-1}.hr^{-1}.flower^{-1})										
**Aromatic compounds**										
Benzaldehyde	635	244.61±199.47	224.35±184.94	200.03±244.46	300.81±244.99	362.54±376.78	403.80±438.70	504.57±539.78	365.42±349.91	291.18±260.23
			CD	D	BC	ABC	ABC	A	AB	BCD
						**0.012**	**0.012**	**2.77*10^{-05}**	**0.002**	
Methyl benzoate	635	35.04±33.39	67.10±84.71	30.53±32.98	33.87±43.02	37.44±44.59	26.68±42.12	32.75±33.58	32.79±36.59	19.27±20.05
			A	BC	BC	AB	BC	BC	AB	C
			**0.003**				**0.039**			**0.004**
Phenylethyl alcohol	635	23.23±35.65	27.05±43.58	29.25±44.32	34.70±45.16	47.12±51.92	16.39±27.12	24.49±38.55	20.14±35.16	24.01±33.91
			BC	BC	AB	A	C	BC	BC	BC
					**0.034**	**3.18*10^{-05}**				
Phenylacetaldehyde	635	586.50±913.11	907.59±1480.09	648.55±910.23	801.30±868.05	1458.44±1497.11	292.24±488.40	700.34±1144.85	391.27±561.36	433.51±682.81
			B	B	B	A	C	BC	BC	BC
						**3.68*10^{-07}**	**0.008**			
p-Anisaldehyde	635	17.75±17.06	20.09±19.67	18.82±21.42	25.38±25.37	30.1±41.11	26.53±33.22	29.30±32.73	20.29±21.43	16.54±34.15

			AB	AB	AB	AB	AB	A	AB	B
								**0.041**		
2-Aminobenzaldehyde	635	428.18±439.37	532.94±599.35	546.68±411.05	326.96±366.17	497.73±452.03	357.98±541.18	477.08±593.74	402.51±451.43	336.25±401.05
			AB	A	B	AB	B	AB	AB	B
				**0.028**						
Methyl salicylate	635	23.84±27.94	23.67±19.44	22.05±23.00	16.60±16.57	22.75±21.46	20.83±21.65	17.57±18.10	29.35±32.84	15.05±13.93
			A	AB	AB	A	AB	AB	A	B
**Terpenoïds**										
β-Pinene	635	8.81±7.10	7.59±7.42	7.83±7.93	7.81±7.86	9.65±18.60	8.16±7.62	7.66±8.12	9.07±9.00	7.79±6.61
			A	A	A	A	A	A	A	A
(E,E)-α-Farnesene	635	239.79±160.79	194.01±133.79	218.42±132.44	309.58±236.96	260.92±174.86	303.01±178.94	285.52±181.65	319.36±187.81	203.01±131.70
			C	BC	ABC	ABC	AB	ABC	A	C
									**0.002**	

**Fatty acid derivatives**										
(Z)-3-Hexen-1-ol-acetate	635	54.10±66.76	59.27±99.98	36.87±44.72	44.15±41.81	47.39±56.38	20.49±23.03	58.48±76.17	59.96±87.40	38.65±44.28
			A	AB	A	A	B	A	A	AB
							**0.002**			
**Nitrogen containing compounds**										
Methyl anthranilate	635	36.66±56.55	67.70±104.66	55.37±73.79	22.90±67.44	42.07±70.12	14.59±25.34	22.47±39.45	24.11±36.16	11.05±21.35
			A	A	CD	AB	CD	BCD	BC	D
			**0.012**		**$3.25^{*}10^{-04}$**		**$7.45^{*}10^{-05}$**	**0.015**		**$2.20^{*}10^{-06}$**
Benzyl nitrile	635	58.69±54.21	103.24±89.28	59.98±54.01	74.88±72.11	65.83±70.26	66.48±75.97	62.18±63.45	51.14±62.74	51.79±61.49
			AB	B	A	AB	AB	A	AB	AB
			**$6.03^{*}10^{-04}$**		**0.042**					
Indole	635	159.78±139.71	231.11±186.65	187.16±116.61	177.96±162.43	199.65±183.53	220.81±223.39	193.95±182.18	169.84±157.44	145.00±148.38
			A	A	A	A	A	A	A	A
			**0.012**	**0.047**						
**Total N-containing volatile compounds**	635	258.13±24.93	402.04±35.18	302.51±24.20	275.74±31.80	307.55±31.80	301.89±35.93	278.60±29.41	245.09±25.79	207.84±25.79
			A	AB	AB	AB	AB	AB	AB	B

			**0.012**							
**Sulphur containing compounds**										
1-Butene-4-isothiocyanate	635	61.06±96.35	49.02±50.39	50.56±56.16	68.77±104.20	88.70±82.93	53.09±56.00	79.95±103.71	58.89±61.12	77.17±101.43
			B	B	AB	A	AB	AB	AB	AB
						**0.006**				
**Total emission**	635	1981.03±1525.46	2514.72±2132.24	2112.10±1443.98	2245.67±1444.41	3171.32±2277.12	1831.09±1455.26	2496.31±1989.04	1954.12±1237.20	1670.26±1310.88
			AB	B	AB	A	B	AB	AB	B
						**$2.68^{*}10^{-04}$**				
Leaf glucosinolates (pg.mg^{-1})										
Glucoraphanin	551	66.36±5.71	54.87±6.20	64.03±5.55	46.14±6.31	71.03±6.73	50.51±4.53	72.55±7.21	49.47±3.94	52.05±4.18
			AB	AB	B	A	AB	A	AB	AB
					**0.015**		**0.018**		**0.033**	**0.048**
Glucoalyssin	566	18.14±3.81	9.68±1.32	15.18±1.79	33.32±8.03	31.74±7.16	28.59±7.72	19.06±3.17	15.76±1.52	23.78±5.36
			B	AB	A	A	A	AB	AB	AB
			**0.035**							

Sinalbin	578	184.55±15.04	160.78±11.17	163.01±12.11	154.22±11.65	166.26±14.79	179.26±15.06	179.68±15.02	165.98±12.44	179.02±14.98
			A	A	A	A	A	A	A	A
Gluconapin	578	10528.85±696.33	8124.47±587.20	9995.91±757.28	8773.15±660.95	10384.45±479.29	10995.42±821.84	10008.11±792.83	10382.91±696.53	11545.03±675.08
			B	AB	B	AB	AB	AB	AB	A
			**0.006**		**0.031**					
Glucobrassicanapin	574	334.43±39.60	254.24±26.29	490.95±58.67	266.52±26.60	301.55±46.35	348.55±42.17	365.26±36.20	518.21±48.75	581.28±65.40
			C	AB	C	C	BC	ABC	A	A
				**0.025**					**0.002**	**9.42*10^{-04}**
Hydroxyglucobrassicin	576	14.22±1.32	12.90±1.03	12.47±1.18	11.35±1.19	8.73±0.93	6.67±0.55	12.69±1.14	10.54±1.17	12.81±1.35
			A	A	A	AB	B	A	AB	A
						**9.07*10^{-04}**	**9.26*10^{-07}**		**0.038**	
Glucobrassicin	576	75.15±10.15	56.11±6.38	74.74±8.45	61.87±9.82	85.27±10.19	53.49±11.68	67.20±9.59	47.53±8.82	69.12±9.30
			AB	AB	AB	B	AB	AB	A	AB
									**0.027**	
Neoglucobrassicin	577	147.36±24.19	173.65±30.83	101.28±23.63	125.62±24.82	137.19±35.60	87.79±24.15	155.78±32.96	92.12±30.18	130.23±25.39
			C	AB	ABC	ABC	A	BC	A	ABC
				**0.006**			**2.56*10^{-04}**		**1.35*10^{-04}**	
Gluconasturtiin	578	500.63±65.87	370.03±51.06	583.61±78.13	331.92±44.33	621.47±61.70	498.42±52.16	596.32±69.87	394.56±39.50	704.49±64.89
			A	ABC	A	BC	ABC	BC	AB	C

					**0.019**					**0.007**
Methoxyglucobrassicin	576	73.73±8.28	86.53±12.70	66.74±8.08	82.22±10.30	67.12±10.75	50.06±6.15	73.27±12.19	32.49±4.62	80.74±9.61
			A	AB	A	AB	AB	AB	B	A
							**0.022**		**$2.21^{*}10^{-05}$**	
**Total glucosinolates (µg.mg^{-1})**	543	11.79+0.78	9.20±0.66	11.29±0.84	10.30±0.87	11.78±0.87	12.19±0.99	11.03±0.81	11.74±0.77	13.10±0.77
			B	AB	AB	AB	AB	AB	AB	A
			**0.006**							

Supplementary Table 8: **Effects of bee-pollination, aphid-herbivory, and their interaction on the evolution of plant morphology, nectar, floral scent, and leaf glucosinolates content in each soil line separately.** Only traits with a significant effect (indicated by Y for "yes") of either bee-pollination, aphid-herbivory, or soil are shown in this table (see Supplementary Table 9 for full statistical values); (+) indicates positive effect (increase) of the factor on trait evolution, whereas (-) indicates a negative effect (decrease) of either factor on plant trait evolution. Only plants of generation 10 were analyzed here.

Trait	N	Pollination (P)		Herbivory (H)		(P) x (H)	
Morphology		*Limestone*	*Tuff*	*Limestone*	*Tuff*	*Limestone*	*Tuff*
Height day 20 (cm)	575		Y (-)				Y
Height day 30 (cm)	575	Y (-)	Y (+)		Y (-)		Y
Time to flower (day)	575	Y (-)	Y (+)				
Leaf size (cm2)	575	Y (-)	Y (+)		Y (-)	Y	
Cumulative branches length (cm)	575			Y (+)	Y (-)	Y	Y
Number of flowering branches	575			Y (+)			
Flower production rate (per day)	575	Y (-)			Y (-)		
Flower number	575	Y (-)	Y (+)		Y (-)	Y	Y
Nectar amount (nL/flower)	564	Y (-)					
Flower diameter (cm)	564	Y (+)	Y (+)				
Petal length (cm)	564	Y (+)	Y (+)				
Petal width (cm)	564	Y (+)	Y (+)	Y (-)			
Sepal length (cm)	564			Y (-)			
Stamen length (cm)	564						
Bioassays							
Number of aphid visits	575		Y (+)				
Number of bee visits	575		Y (+)		Y (-)		
Floral scent							
Benzaldehyde	564			Y (-)	Y (+)		Y
Methyl benzoate	564	Y (+)				Y	Y
Phenylethyl alcohol	564			Y (-)			
Phenylacetaldehyde	564		Y (-)	Y (-)		Y	
p-Anisaldehyde	564				Y (+)		
2-Aminobenzaldehyde	564	Y (-)		Y (+)			
Methyl salicylate	564		Y (+)			Y	
(E,E)-α-Farnesene	564		Y (+)	Y (-)			Y
(Z)-3-Hexen-1-ol-acetate	564				Y (-)		Y
Methyl anthranilate	564	Y (-)		Y (+)		Y	Y
Benzyl nitrile	564	Y (+)					
1-Butene-4-isothiocyanate	564			Y (-)			
Total emission	564					Y	Y
Leaf glucosinolates							
Glucoraphanin	485	Y (-)	Y (-)		Y (+)		
Glucoalyssin	500			Y (-)			
Gluconapin	512	Y (-)					
Glucobrassicanapin	509	Y (-)		Y (+)	Y (-)	Y	
Hydroxyglucobrassicin	510		Y (-)	Y (+)			
Glucobrassicin	510	Y (-)	Y (-)				
Neoglucobrassicin	511	Y (-)	Y (-)			Y	

Gluconasturtiin	512	Y (-)	Y (-)				Y
Methoxyglucobrassicin	511		Y (-)				
Total glucosinolates	477	Y (-)					

Supplementary Table 9: **Effects of bee-pollination, aphid-herbivory, and their interaction on the evolution of plant morphology, nectar, floral scent, and leaf glucosinolates content in each soil line separately.** Significance between pollination and herbivory treatments in each soil line was determined using two-sided linear mixed models (LMM) with individual traits as dependent variable, pollination, herbivory, and their interaction as fixed factors, and replicate as random factors. Bold indicates significant factor effects (P<0.05). (+) indicates positive effect (increase) of either bees or herbivory on plant traits evolution whereas (-) indicate a negative effect (decrease). Only plants of generation 10 were analyzed here.

			Limestone lines				Tuff lines			
Traits	Factor	df	N	χ^2	P	sign	N	χ^2	P	sign
Morphology										
Heigth at day 20 (cm)	Pollination (P)	1	287	1.17	0.280		288	**51.48**	**$7.23^{*}10^{-13}$**	-
	Herbivory (H)	1		2.03	0.154			1.54	0.214	
	Replicate	1		0.01	0.921			0.16	0.693	
	(P) x (H)	1		1.46	0.227			**4.91**	**0.027**	
Heigth at day 30 (cm)	Pollination (P)	1	287	**16.06**	**$6.14^{*}10^{-05}$**	-	288	**4.41**	**0.036**	+
	Herbivory (H)	1		1.67	0.196			**26.48**	**$2.67^{*}10^{-07}$**	-
	Replicate	1		0.12	0.731			0.04	0.845	
	(P) x (H)	1		**4.75**	**0.029**			**7.57**	**0.006**	
Time to flower (day)	Pollination (P)	1	287	**10.52**	**0.001**	-	288	**40.57**	**$1.90^{*}10^{-10}$**	+
	Herbivory (H)	1		0.43	0.510			0.29	0.593	
	Replicate	1		0.27	0.870			0.02	0.895	
	(P) x (H)	1		0.13	0.715			0.64	0.422	
Leaf size (cm^2)	Pollination (P)	1	287	**5.16**	**0.023**	-	288	**39.32**	**$3.59^{*}10^{-10}$**	+
	Herbivory (H)	1		0.24	0.624			**8.20**	**0.007**	-
	Replicate	1		0.00	0.958			0.00	0.962	
	(P) x (H)	1		**4.66**	**0.031**			0.21	0.650	
Cumulative branches length (cm)	Pollination (P)	1	287	0.08	0.777		288	0.82	0.366	
	Herbivory (H)	1		**4.65**	**0.031**	+		**4.98**	**0.026**	-
	Replicate	1		0.01	0.931			0.01	0.933	
	(P) x (H)	1		**5.66**	**0.017**			**4.67**	**0.031**	
Number of flowering branches	Pollination (P)	1	287	2.75	0.097		288	3.07	0.080	
	Herbivory (H)	1		**3.91**	**0.048**	+		0.04	0.841	
	Replicate	1		0.10	0.747			0.01	0.928	
	(P) x (H)	1		2.58	0.108			0.24	0.625	
Flower production rate (per day)	Pollination (P)	1	287	5.73	**0.017**	-	288	0.65	0.421	
	Herbivory (H)	1		3.37	0.066			**9.36**	**0.002**	-

	Replicate	1		0.53	0.466			0.03	0.858	
	(P) x (H)	1		3.81	0.051			0.34	0.562	
Number of flowers	Pollination (P)	1	287	**9.69**	**0.002**	-	288	**6.16**	**0.013**	+
	Herbivory (H)	1		1.96	0.162			**9.80**	**0.002**	-
	Replicate	1		0.09	0.760			0.01	0.905	
	(P) x (H)	1		**6.96**	**0.008**			**4.66**	**0.031**	
Nectar amount (nL/flower)	Pollination (P)	1	281	**5.75**	**0.016**	-	282	1.87	0.171	
	Herbivory (H)	1		0.35	0.553			3.04	0.081	
	Replicate	1		0.01	0.910			0.01	0.917	
	(P) x (H)	1		0.61	0.434			0.04	0.834	
Flower diameter (cm)	Pollination (P)	1	283	**17.71**	**$2.58^{*}10^{-04}$**	+	281	**27.44**	**$1.62^{*}10^{-07}$**	+
	Herbivory (H)	1		3.27	0.071			0.21	0.648	
	Replicate	1		0.45	0.503			0.03	0.862	
	(P) x (H)	1		0.07	0.787			2.08	0.149	
Petal length (cm)	Pollination (P)	1	283	**7.03**	**0.008**	+	281	**23.31**	**$1.38^{*}10^{-06}$**	+
	Herbivory (H)	1		0.92	0.336			0.73	0.390	
	Replicate	1		1.16	0.280			0.36	0.547	
	(P) x (H)	1		0.59	0.444			0.87	0.350	
Petal width (cm)	Pollination (P)	1	283	**27.03**	**$2.00^{*}10^{-07}$**	+	281	**7.07**	**0.008**	+
	Herbivory (H)	1		**18.94**	**$1.35^{*}10^{-05}$**	-		2.35	0.125	
	Replicate	1		1.14	0.286			0.00	0.948	
	(P) x (H)	1		0.90	0.343			0.45	0.501	
Sepal length (cm)	Pollination (P)	1	283	0.04	0.836		281	1.98	0.160	
	Herbivory (H)	1		**17.08**	**$3.58^{*}10^{-05}$**	-		0.29	0.591	
	Replicate	1		0.01	0.918			0.24	0.624	
	(P) x (H)	1		0.25	0.618			2.60	0.107	
Style length (cm)	Pollination (P)	1	283	0.26	0.608		281	0.83	0.361	
	Herbivory (H)	1		0.08	0.774			**4.23**	**0.040**	+
	Replicate	1		0.09	0.765			1.70	0.193	
	(P) x (H)	1		0.08	0.782			**4.61**	**0.032**	
Stamen length (cm)	Pollination (P)	1	283	0.17	0.678		281	0.10	0.754	
	Herbivory (H)	1		0.45	0.503			1.14	0.286	
	Replicate	1		**4.25**	**0.039**			0.63	0.428	
	(P) x (H)	1		0.19	0.663			**6.05**	**0.014**	
Herkogamy	Pollination (P)	1	283	0.26	0.609		281	0.17	0.681	
	Herbivory (H)	1		0.22	0.642			3.17	0.075	
	Replicate	1		0.00	0.962			1.08	0.300	
	(P) x (H)	1		0.72	0.396			**4.12**	**0.043**	
Bioassays										
Number of aphid visits	Pollination (P)	1	288	1.66	0.197		288	**0.18**	**0.023**	+

	Herbivory (H)	1		0.33	0.566			1.43	0.232	
	Replicate	1		2.75	0.097			**4.77**	**0.029**	
	(P) x (H)	1		2.91	0.088			1.65	0.199	
Number of bee visits	Pollination (P)	1	288	0.80	0.371		288	6.55	**0.010**	+
	Herbivory (H)	1		0.56	0.455			4.19	**0.041**	-
	Replicate	1		0.08	0.773			1.46	0.226	
	(P) x (H)	1		2.33	0.127			3.52	0.061	
Floral scent (pg.l^{-1}.hr^{-1}.flower^{-1})										
**Aromatic compounds**										
Benzaldehyde	Pollination (P)	1	282	0.15	0.702		282	0.01	0.940	
	Herbivory (H)	1		**19.09**	**$1.25^{*}10^{-05}$**	**-**		**4.79**	**0.003**	+
	Replicate	1		0.35	0.557			0.30	0.584	
	(P) x (H)	1		3.57	0.059			**6.67**	**0.010**	
Methyl benzoate	Pollination (P)	1	282	**5.04**	**0.025**	+	282	0.96	0.327	
	Herbivory (H)	1		2.76	0.097			0.03	0.867	
	Replicate	1		0.01	0.932			0.28	0.597	
	(P) x (H)	1		**10.01**	**0.002**			**15.81**	**$6.99^{*}10^{-05}$**	
Phenylethyl alcohol	Pollination (P)	1	282	2.09	0.148		282	2.65	0.104	
	Herbivory (H)	1		**9.28**	**0.002**	-		0.95	0.330	
	Replicate	1		0.20	0.652			0.33	0.568	
	(P) x (H)	1		2.03	0.154			0.19	0.664	
Phenylacetaldehyde	Pollination (P)	1	282	2.80	0.094		282	**3.88**	**0.049**	**-**
	Herbivory (H)	1		**11.74**	**$6.13^{*}10^{-04}$**	+		0.57	0.452	
	Replicate	1		0.29	0.593			0.33	0.567	
	(P) x (H)	1		**9.67**	**0.002**			3.06	0.080	
p-Anisaldehyde	Pollination (P)	1	282	0.22	0.639		282	1.51	0.219	
	Herbivory (H)	1		3.76	0.053			**8.76**	**0.003**	+
	Replicate	1		1.57	0.210			0.94	0.333	
	(P) x (H)	1		0.11	0.735			3.56	0.059	
2-Aminobenzaldehyde	Pollination (P)	1	282	**6.25**	**0.012**	-	282	0.19	0.666	
	Herbivory (H)	1		**4.94**	**0.026**	+		0.36	0.551	
	Replicate	1		0.00	0.992			0.06	0.804	
	(P) x (H)	1		0.27	0.600			2.95	0.086	
Methyl salicylate	Pollination (P)	1	282	0.35	0.556		282	**8.50**	**0.004**	+
	Herbivory (H)	1		1.94	0.164			0.01	0.915	
	Replicate	1		0.19	0.664			0.14	0.709	
	(P) x (H)	1		**8.59**	**0.003**			1.53	0.216	
**Terpenoids**										
β-Pinene	Pollination (P)	1	282	0.00	0.858		282	0.49	0.483	
	Herbivory (H)	1		0.01	0.917			1.02	0.312	
	Replicate	1		0.01	0.909			0.21	0.644	

	(P) x (H)	1		0.59	0.443			0.23	0.643	
(E,E)-α-Farnesene	Pollination (P)	1	282	0.21	0.648		282	**11.08**	**$8.72^{*}10^{-04}$**	+
	Herbivory (H)	1		**10.39**	**0.001**	-		1.73	0.188	
	Replicate	1		0.00	0.947			0.85	0.357	
	(P) x (H)	1		0.15	0.698			**7.43**	**0.006**	
**Fatty acid derivatives**										
(Z)-3-Hexen-1-ol-acetate	Pollination (P)	1	282	2.88	0.090		282	3.65	0.056	
	Herbivory (H)	1		0.36	0.548			**5.09**	**0.024**	**-**
	Replicate	1		0.11	0.740			0.07	0.790	
	(P) x (H)	1		0.74	0.388			**19.83**	**$8.48^{*}10^{-06}$**	
**Nitrogen containing compounds**										
Methyl anthranilate	Pollination (P)	1	282	**4.33**	**0.038**	-	282	1.35	0.245	
	Herbivory (H)	1		**30.22**	**$3.85^{*}10^{-04}$**	+		0.01	0.932	
	Replicate	1		0.03	0.867			0.42	0.516	
	(P) x (H)	1		**11.87**	**$5.72^{*}10^{-04}$**			**11.34**	**$7.57^{*}10^{-06}$**	
Benzyl nitrile	Pollination (P)	1	282	**6.68**	**0.010**	+	282	0.14	0.711	
	Herbivory (H)	1		0.56	0.454			0.67	0.412	
	Replicate	1		0.88	0.348			0.03	0.864	
	(P) x (H)	1		2.81	0.094			0.20	0.657	
Indole	Pollination (P)	1	282	0.28	0.597		282	3.61	0.057	
	Herbivory (H)	1		3.16	0.075			1.18	0.277	
	Replicate	1		0.02	0.899			0.29	0.581	
	(P) x (H)	1		1.92	0.165			0.25	0.616	
**Total N-containing volatile compounds**	Pollination (P)	1	282	0.09	0.767		282	3.09	0.079	
	Herbivory (H)	1		3.16	0.075			0.86	0.354	
	Replicate	1		0.16	0.688			0.26	0.612	
	(P) x (H)	1		2.89	0.090			0.91	0.341	
**Sulfphur containing compounds**										
1-Butene-4-isothiocyanate	Pollination (P)	1	282	1.36	0.243		282	1.86	0.173	
	Herbivory (H)	1		**11.06**	**$8.82^{*}10^{-04}$**	-		0.63	0.548	
	Replicate	1		0.11	0.744			0.16	0.689	
	(P) x (H)	1		0.98	0.323			0.68	0.410	
**Total emission**	Pollination (P)	1	282	0.80	0.371		282	0.07	0.789	
	Herbivory (H)	1		3.00	0.083			1.22	0.270	
	Replicate	1		0.10	0.752			0.13	0.723	
	(P) x (H)	1		**6.94**	**0.008**			**7.41**	**0.006**	
Leaf glucosinolates ($pg.mg^{-1}$)										
Glucoraphanin	Pollination (P)	1	242	**7.60**	**0.006**	-	243	**5.16**	**0.023**	-
	Herbivory (H)	1		0.02	0.883			**4.49**	**0.034**	+
	Replicate	1		0.14	0.704			0.00	0.947	
	(P) x (H)	1		1.63	0.202			3.49	0.062	

Glucoalyssin	Pollination (P)	1	252	0.16	0.691		248	0.01	0.940	
	Herbivory (H)	1		**13.26**	**$2.71^{*}10^{-04}$**	-		0.98	0.322	
	Replicate	1		0.02	0.878			1.40	0.237	
	(P) x (H)	1		0.42	0.519			3.28	0.070	
Sinalbin	Pollination (P)	1	257	0.28	0.599		255	0.22	0.641	
	Herbivory (H)	1		0.06	0.804			0.25	0.617	
	Replicate	1		0.05	0.815			0.00	0.964	
	(P) x (H)	1		0.14	0.708			0.17	0.681	
Gluconapin	Pollination (P)	1	257	**6.25**	**0.012**	-	255	0.41	0.522	
	Herbivory (H)	1		0.25	0.615			1.27	0.260	
	Replicate	1		0.00	0.992			0.05	0.826	
	(P) x (H)	1		0.08	0.779			3.03	0.082	
Glucobrassicanapin	Pollination (P)	1	257	**9.63**	**0.002**	-	252	0.58	0.446	
	Herbivory (H)	1		**5.28**	**0.022**	+		**15.51**	**$8.19^{*}10^{-05}$**	-
	Replicate	1		0.00	0.997			0.04	0.851	
	(P) x (H)	1		**7.11**	**0.008**			0.01	0.910	
Hydroxyglucobrassicin	Pollination (P)	1	256	2.01	0.156		254	**13.61**	**$2.25^{*}10^{-04}$**	-
	Herbivory (H)	1		**5.99**	**0.014**	+		3.29	0.070	
	Replicate	1		0.12	0.725			0.00	0.975	
	(P) x (H)	1		1.03	0.309			2.91	0.088	
Glucobrassicin	Pollination (P)	1	256	**6.00**	**0.014**	-	254	**4.21**	**0.040**	-
	Herbivory (H)	1		0.69	0.406			0.03	0.873	
	Replicate	1		0.03	0.871			0.09	0.763	
	(P) x (H)	1		0.09	0.759			0.15	0.699	
Neoglucobrassicin	Pollination (P)	1	256	**4.75**	**0.029**	-	255	**16.77**	**$4.21^{*}10^{-05}$**	-
	Herbivory (H)	1		0.28	0.598			0.66	0.418	
	Replicate	1		0.10	0.750			0.01	0.930	
	(P) x (H)	1		**8.19**	**0.004**			1.03	0.309	
Gluconasturtiin	Pollination (P)	1	257	**20.55**	**$5.82^{*}10^{-}04$**	-	255	**13.30**	**$2.65^{*}10^{-04}$**	-
	Herbivory (H)	1		0.10	0.749			0.03	0.861	
	Replicate	1		2.40	0.121			0.08	0.778	
	(P) x (H)	1		1.09	0.296			**4.32**	**0.038**	
Methoxyglucobrassicin	Pollination (P)	1	257	2.51	0.113		254	**19.04**	**$1.28^{*}10^{-05}$**	-
	Herbivory (H)	1		0.06	0.809			0.34	0.559	
	Replicate	1		0.73	0.392			0.11	0.742	
	(P) x (H)	1		0.00	0.946			2.70	0.100	
**Total glucosinolates**	Pollination (P)	1	238	**5.19**	**0.023**	-	239	0.58	0.447	
	Herbivory (H)	1		0.67	0.414			2.37	0.124	
	Replicate	1		0.02	0.900			0.06	0.808	
	(P) x (H)	1		0.25	0.620			2.56	0.110	

Supplementary Table 10: Rotated loadings of the morphological, scent and glucosinolate variables on the principal components calculated from of *Brassica rapa* data in generation one and ten combined. The highest loading of each variable is indicated in bold. Only principal components with eigenvalues >1 were retained. Positive loadings indicate positive correlations of PCs scores and variables, whereas negative loadings values indicate negative correlations.

	PC1	PC2	PC3	PC4	PC5	PC6	PC7	PC8	PC9	PC10	PC11	PC12
Eigenvalues	5.93	4.23	3.75	2.98	2.63	1.69	1.56	1.43	1.35	1.19	1.08	1.01
Variance explained (%)	14.83	10.58	9.36	7.45	6.58	4.22	3.90	3.57	3.39	2.98	2.71	2.53
	Rotated loadings on retained components											
Height day 20 (cm)	0.07	0.21	-0.12	**-0.82**	-0.02	0.09	0.10	0.13	0.03	0.00	0.04	-0.04
Height day 30 (cm)	0.00	**0.59**	0.25	-0.01	0.20	0.05	0.04	-0.02	0.07	-0.22	-0.26	-0.07
Leaf size (cm2)	0.01	0.39	0.16	**0.55**	0.29	0.00	-0.01	-0.09	-0.02	-0.03	-0.24	-0.12
Time to flower (day)	-0.02	-0.04	0.13	**0.86**	0.04	-0.01	-0.09	-0.11	0.02	-0.06	-0.03	0.08
Cumulative branches length (cm)	0.04	**0.75**	-0.14	-0.42	0.00	0.05	-0.01	0.07	0.04	0.08	0.03	-0.03
Number of flowering branches	0.04	**0.75**	-0.17	-0.38	-0.02	-0.02	0.03	0.13	-0.03	0.13	0.14	0.01
Flower production rate (per day)	0.03	**0.77**	0.13	0.42	0.11	-0.10	-0.08	-0.02	-0.04	-0.12	-0.06	0.08
Flower number	0.05	**0.89**	0.11	0.08	0.10	-0.11	-0.05	0.01	-0.05	-0.11	-0.07	0.03
Nectar amount (nL/flower)	-0.04	0.15	**0.48**	0.20	0.21	-0.05	0.06	0.02	-0.02	-0.05	-0.15	0.06
Flower diameter (cm)	0.06	0.07	-0.10	0.01	**0.85**	0.06	0.01	0.08	0.03	-0.03	-0.10	0.10
Petal length (cm)	0.09	0.09	-0.01	0.06	**0.90**	0.15	-0.04	-0.02	0.04	0.01	-0.04	0.03
Petal width (cm)	0.07	0.03	-0.01	0.08	**0.80**	-0.06	0.07	0.09	-0.04	-0.01	-0.06	-0.04
Sepal length (cm)	0.15	0.04	-0.13	0.32	**0.51**	0.23	0.06	-0.03	-0.14	0.18	0.08	-0.08
Stamen length (cm)	0.16	0.08	0.14	-0.11	**0.66**	-0.03	-0.21	-0.16	0.09	-0.10	0.10	-0.03
Style length (cm)	0.00	-0.06	0.09	-0.10	0.39	**0.81**	-0.08	-0.01	0.03	-0.04	0.04	-0.04
Herkogamy (cm)	-0.06	-0.06	0.02	-0.02	-0.07	**0.93**	0.03	0.03	-0.09	0.02	-0.05	0.02
Benzaldehyde	0.22	0.01	0.10	0.07	-0.02	-0.01	0.38	0.04	0.17	0.03	0.00	**0.72**
Methyl benzoate	0.13	0.00	-0.06	-0.14	0.06	0.00	0.30	0.04	**0.77**	0.02	-0.01	0.22
Phenylethyl alcohol	**0.83**	0.02	0.00	-0.02	0.16	-0.06	0.37	0.01	-0.04	-0.04	0.00	-0.09
Phenylacetaldehyde	**0.84**	-0.05	0.01	-0.08	0.13	-0.08	0.28	0.07	0.04	-0.02	0.01	-0.01
p-Anisaldehyde	0.26	0.00	0.09	-0.01	0.01	-0.03	0.04	-0.02	0.17	0.05	-0.06	**0.79**
2-Aminobenzaldehyde	**0.73**	0.11	0.10	0.01	0.01	-0.02	-0.09	0.04	0.33	-0.03	-0.01	0.27
Methyl salicylate	0.25	-0.02	0.04	0.14	-0.01	-0.05	0.24	0.02	**0.75**	0.04	-0.02	0.15
β-Pinene	0.00	0.05	-0.30	**0.40**	-0.03	-0.05	0.14	0.09	0.13	0.36	0.06	-0.30
(E,E)-α-Farnesene	**0.45**	0.09	0.00	0.14	0.06	0.05	0.45	-0.01	0.32	0.04	0.00	0.38
(Z)-3-Hexen-1-ol-acetate	0.16	-0.06	-0.07	-0.24	-0.09	0.01	**0.73**	0.02	0.17	0.01	0.00	0.07
Methyl anthranilate	0.34	-0.03	-0.04	-0.03	-0.02	-0.04	-0.10	0.02	**0.84**	-0.01	0.01	-0.01
Benzyl nitrile	**0.75**	0.03	0.00	0.00	0.12	0.03	0.03	0.00	0.38	0.00	0.00	0.25
1-Butene-4-isothiocyanate	0.08	-0.03	-0.04	0.01	-0.02	-0.03	**0.80**	-0.08	0.08	-0.04	0.07	0.11
Indole	**0.71**	0.05	0.01	-0.04	0.11	0.06	-0.15	-0.01	0.25	-0.02	-0.08	0.24
Glucoraphanin	0.01	-0.11	0.31	-0.16	-0.03	0.04	-0.04	-0.04	0.01	**0.75**	0.08	-0.01
Glucoalyssin	-0.10	-0.08	0.32	0.05	0.00	-0.03	-0.01	-0.11	0.01	**0.73**	-0.06	0.11
Sinalbin	0.03	-0.02	**0.69**	-0.06	-0.05	0.07	-0.08	0.07	0.04	0.10	0.26	0.04
Gluconapin	0.07	0.00	**0.84**	-0.01	-0.06	0.11	-0.01	-0.09	-0.01	0.18	0.05	0.04
Glucobrassicanapin	-0.03	0.11	**0.81**	0.16	-0.08	-0.01	0.01	-0.02	-0.06	0.08	-0.12	0.00
Hydroxyglucobrassicin	0.00	0.10	0.07	-0.07	0.05	-0.02	-0.03	**0.88**	0.05	-0.08	0.03	-0.08

Glucobrassicin	0.01	0.04	0.12	-0.03	-0.06	0.07	0.02	0.01	-0.01	0.07	**0.81**	0.02
Neoglucobrassicin	0.09	-0.01	-0.13	-0.18	-0.04	0.04	-0.03	**0.85**	0.01	-0.03	0.13	0.09
Gluconasturtiin	0.05	-0.06	**0.63**	0.13	-0.03	-0.07	-0.10	-0.03	0.01	0.23	0.25	0.06
Methoxyglucobrassicin	-0.08	-0.15	0.07	-0.09	0.00	-0.09	0.05	0.14	0.00	-0.05	**0.73**	-0.10

Supplementary Table 11: Physical, chemical, and mineralogical features of each soil used in this study. Soil physics and chemistry was analyzed by the INRA laboratories following the SOL-1031 protocol [2]. This method used an acid mixed (HF-LCO4), which digests soil clay and thus quantifies both available and unavailable nutrients to plants.

			Limestone	Tuff
Physical parameters	Clay	%	9.30	6.70
	Fine Silt		17.40	13.70
	Coarse Silt		6.90	12.80
	Fine Sand		14.20	20.80
	Coarse Sand		52.20	46.00
Chemical Parameters	N	g/kg	2.57	1.06
	P	g/kg	3.61	1.79
	Org. Carbon	g/kg	28.40	12.50
	Soil organic matter	g/kg	49.10	21.60
	C/N		11.00	11.80
	Cu	g / 100 g	0.48	0.35
	Zn	g / 100 g	0.77	0.91
	CaCO3	g / 100 g	19.40	0.60
	Si	g / 100 g	17.00	26.50
	Ca	meq / 100 g	5.94	1.12
	Mg	meq / 100 g	0.91	0.26
	K	meq / 100 g	0.75	1.56
	Na	meq / 100 g	0.37	0.96
	Fe	meq / 100 g	5.59	4.05
	Mn	meq / 100 g	9.93	11.71
	Al	meq / 100 g	0.31	8.53

Supplementary References

1 Lenth, R. V. Estimated marginal means, aka least-squares means [R Package Emmeans Version 1.6. 0]. Comprehensive R Archive Network (CRAN). (2021).

2 Ciesielski, H., Proix, N. & Sterckeman, T. Détermination des incertitudes liées à une méthode de mise en solution des sols et sédiments par étude interlaboratoire. *Analusis* **25**, 188-192 (1997).
