# Supplementary material for: Bee-pollination promotes rapid divergent evolution in plants growing in different soils
Source: Nat Commun. 2024 Mar 27;15:2703. doi: 10.1038/s41467-024-46841-4 (PMC10973342; doi:10.1038/s41467-024-46841-4)
Supplement: Supplementary file 3 — Description of Additional Supplementary Files [file 41467_2024_46841_MOESM3_ESM.pdf]

Description of Additional Supplementary Files

File Name: Supplementary Data 1**. Dataset.**
Description: Traits values among first generation plants and those having evolved with or without aphid-herbivory, with or without bee-pollination and either in limestone or tuff soil. G1: plants of generation one. LHB: limestone line plants (L) growing with aphid-herbivory (H) and bee-pollination (B). LHH: limestone line plants (L) growing with aphid-herbivory (H) and hand-pollination (H). LNHB: limestone line plants (L) growing without herbivory (NH) and bee-pollination (B). LNHH: limestone line plants (L) growing without herbivory (NH) and hand-pollination (H). THB: tuff line plants (T) growing with aphid-herbivory (H) and bee-pollination (B). THH: tuff line plants (T) growing with aphid-herbivory (H) and hand-pollination (H). TNHB: tuff line plants (T) growing without herbivory (NH) and bee-pollination (B). TNHH: tuff line plants (T) growing without herbivory (NH) and hand-pollination (H).

File Name: Supplementary Data 2**. Dataset.**
Description: F values for each specific trait, assessing the phenotypic divergence in plants of different soil types in generation 10. ANOVA was done for 33 individual traits in all treatment groups and replicates separately, using "soil" as independent factor. HB plants growing with aphid-herbivory (H) and bee-pollination (B). HH: plants growing with aphid-herbivory (H) and hand-pollination (H). NHB: plants growing without herbivory (NH) and bee-pollination (B). NHH: plants growing without herbivory (NH) and hand-pollination (H). Traits were categorized in three different classes: morphology (M), floral scent emission (S), and glucosinolates (G).

File Name: Supplementary Data 3**. Dataset.**
Description: Principal components scores and traits values used for multivariate linear discriminant function analyses among first generation plants and those having evolved with or without aphid-herbivory, with or without bee-pollination and either in limestone or tuff soil. G1: plants of generation one. LHB: limestone line plants (L) growing with aphid-herbivory (H) and bee-pollination (B). LHH: limestone line plants (L) growing with aphid-herbivory (H) and hand-pollination (H). LNHB: limestone line plants (L) growing without herbivory (NH) and bee-pollination (B). LNHH: limestone line plants (L) growing without herbivory (NH) and hand-pollination (H). THB: tuff line plants (T) growing with aphid-herbivory (H) and bee-pollination (B). THH: tuff line plants (T) growing with aphid-herbivory (H) and hand-pollination (H). TNHB: tuff line plants (T) growing without herbivory (NH) and bee-pollination (B). TNHH: tuff line plants (T) growing without herbivory (NH) and hand-pollination (H).

File Name: Supplementary Data 4**. Dataset.**
Description: Haldane's values for each specific trait, among plants having evolved with or without aphid-herbivory, with or without bee-pollination and either in limestone or tuff soil. G1: plants of generation one. LHB: limestone line plants (L) growing with aphid-herbivory (H) and bee-pollination (B). LHH: limestone line plants (L) growing with aphid-herbivory (H) and hand-pollination (H). LNHB: limestone line plants (L) growing without herbivory (NH) and bee-pollination (B). LNHH: limestone line plants (L) growing without herbivory (NH) and hand-pollination (H). THB: tuff line plants (T) growing with aphid-herbivory (H) and bee-pollination (B). THH: tuff line plants (T) growing with aphid-herbivory (H) and hand-pollination (H). TNHB: tuff line plants (T) growing without herbivory (NH) and bee-

pollination (B). TNHH: tuff line plants (T) growing without herbivory (NH) and hand-pollination (H). Traits were categorized in three different classes: morphology (M), floral scent emission (S), and glucosinolates (G).

File name: Supplementary Data 5. **Dataset.**
Description: Reproductive seed sets of intra-replicate and inter-replicate crossings conducted in generation eight (F0), and trait values of the offspring issued from these crossings (F1). LHB: limestone line plants (L) growing with aphid-herbivory (H) and bee-pollination (B). LHH: limestone line plants (L) growing with aphid-herbivory (H) and hand-pollination (H). LNHB: limestone line plants (L) growing without herbivory (NH) and bee-pollination (B). LNHH: limestone line plants (L) growing without herbivory (NH) and hand-pollination (H). THB: tuff line plants (T) growing with aphid-herbivory (H) and bee-pollination (B). THH: tuff line plants (T) growing with aphid-herbivory (H) and hand-pollination (H). TNHB: tuff line plants (T) growing without herbivory (NH) and bee-pollination (B). TNHH: tuff line plants (T) growing without herbivory (NH) and hand-pollination (H).

File Name: Supplementary Data 6**. Dataset.**
Description: Traits values of plants growing in different soil (L = limestone, T= Tuff), and in different soil treatment (fertilizer: with use of fertilizer, nofertilizer: without use of fertilizer, standardized: grown in standardized soil, which provides optimal conditions for cultivation).
